# Ex-Vapers’ Perspectives on Helpful and Unhelpful Influences During Their Quit Journeys

**DOI:** 10.3390/ijerph22071073

**Published:** 2025-07-04

**Authors:** Mohammed Al-Hamdani, Courtney McKay, Katelynn Carter-Rogers, Steven Smith

**Affiliations:** 1 Department of Public Health, College of Health Science, QU Health, Qatar University, Doha 2713, Qatar; malhamdani@qu.edu.qa; 2 Department of Psychology, Saint Mary’s University, 923 Robie Street, Halifax, NS B3H 3C3, Canada; courtney.mckay@smu.ca; 3 Department of Management, St. Francis Xavier University, 4130 University Ave, Antigonish, NS B2G 2W5, Canada; kcarter@stfx.ca

**Keywords:** ex-vapers, quitting behaviors, policy change, vaping cessation

## Abstract

There is limited understanding of what influences vaping cessation, especially as vaping regulations change, and different jurisdictions have different regulations. This study involves 281 ex-vapers (16–24 years) from Nova Scotia, Canada. A content analysis was used to understand and compare youth and young adults’ (YA) experiences of quitting vaping. Both helpful and unhelpful factors for quitting vaping were identified; each category had five themes and twenty-one sub-themes. Helpful factors were consistent across both age categories and included planned and unplanned vaping control interventions, health concerns, social support, evidence-based support, and unassisted quitting methods. Similarly, the five themes identified as unhelpful factors were consistent for both age groups: negative personal implications, negative social influences, planned and unplanned vaping control interventions, the side effects of previous use, and simultaneous and alternative substance use. Policies that limit access and raise awareness about lung health and well-being can help youth quit vaping. For YAs, increasing awareness about social support and health concerns is crucial. Raising e-cigarette costs and reducing vaping normalization supports quitting for YAs. Stress reduction and training to handle social pressure could aid youth, while YAs might benefit from treatment for other substance use to help with nicotine quitting.

## 1. Introduction

E-cigarettes are popular with an estimated 68 million users worldwide [1]. The growth of e-cigarette use is attributed to many factors including acquisitions of e-cigarette companies by “Big Tobacco” (which has a history of increasing product addictiveness), targeting young populations, and maximizing profits at the expense of public health [2]. This growth has stimulated research on the topic with respect to liked and disliked aspects of the products from the consumer’s perspective [3], its association with tobacco use [4], social factors that influence vaping behavior [5], and strategies for quitting its use [6]. Canadian youth and young adults (YAs) report a higher prevalence of use than older age groups [7], and most youth that vape are non-smokers [8], making the issue of paramount importance to public health. The social factors that influence vaping can be mapped into the socio-ecological model which depicts individual-level factors (e.g., demographics), interpersonal factors (e.g., the characteristics of friends), organizational and community factors (e.g., school and access to shops), and social and police factors (e.g., policies) [9].

At an individual level, youth and YAs vape for different reasons, including flavors in vaping products and experiencing the psychoactive effects of nicotine [10]. For instance, youth perceive a higher ease of use for e-cigarettes that contain flavors relative to those that do not [11]. The individual-level influences that vapers gravitate towards vary by age and sex; for example, male youth vapers consider the rush experienced from nicotine-based e-cigarettes to be their best aspect for use relative to male YAs [12]. Further, the main reason for vaping among males is their perception that the products are less harmful than cigarettes. For females, the major reason for vaping is the tendency of vaping to be less harmful to others [13].

At the interpersonal level, youth report that decisions to vape or not vape are affected by approval from peers, social identity, and feeling cool, all of which promote vaping [14]. Among YAs in college populations, a higher number of peers who vape was associated with increased offers to vape and a higher likelihood to vape [15]. Interpersonal factors have differing influences on vapers depending on sex and tobacco use status—males who currently use tobacco experience more pressure to vape relative to female counterparts and female vapers who currently use tobacco are more likely to report being influenced by their social circle on social media [16]. Interpersonal level influences also vary by ethnicity; for instance, Whites and Hispanics report more peer influence to vape [17].

Organization and community factors that influence use include access to retail products: youth report access to vaping products from online purchases, social sourcing, or buying used products [18]. Schools that ban e-cigarettes have lower odds of students using e-cigarettes relative to those that do not [19]. The social and policy factors that influence vaping behavior are mostly regulatory in nature. E-cigarette use is lower in jurisdictions that implement taxes compared to those that do not [20]. Increasing the minimum age for use to 21 is associated with no change in use prevalence for youth and YAs, while a lack of such policy measures is associated with an increase in prevalence [20]. Jurisdictions with comprehensive vaping control policies that include indoor vaping bans, taxes, and increasing the minimum age to 21 have a lower prevalence of vaping relative to those without them [19].

Ex-vapers cite fear of getting addicted, social unacceptability, failure to succeed in quitting tobacco use through vaping, and cost as some reasons for discontinuing use [21]. Further, nicotine e-cigarette users cite concerns over one’s health and distractions from performing hobbies and activities as reasons for intending to quit [22]. The strategies for quitting e-cigarette use include individual-level approaches such as self-restriction and eliminating triggers, and social-level factors such as eliminating social influences and support systems [23]. However, the supportive and unsupportive forces that influence use among youth and YAs following the implementation of vaping control policies has not been studied. Such influences are important to understand in order to better develop interventions and policies to address vaping among these groups, given their disproportionately higher vaping prevalence relative to older populations (e.g., [7]).

The province of Nova Scotia (NS) has implemented vaping control policies including flavor bans, retail license requirements, and nicotine caps, most prior to September 2020 [24]. Studying the beliefs and behaviors of ex-vapers who experienced these policies prior to quitting or during maintenance is important as it can help elucidate the role of policies and other factors influencing their decision to quit. In this study, we examined what or who ex-vapers in NS found useful in supporting them to quit vaping and, on the contrary, what or who ex-vapers found unhelpful in their quit journey. The policies were implemented between April and 1 September 2020. Details about the policies and their timelines can be found in Kennedy et al., 2022, [25], and the sample in this study were ex-vapers who completed the study shortly after the final policy implementation. The objective of this study was to learn about the helpful and unhelpful factors that are associated with the quitting journeys of a sample of NS vapers, shortly after the implementation of vaping control policies in the province.

## 2. Methods

The participants in this study were recruited using paid boosted ads on social media platforms in Fall 2021. The ads contained a link to a Qualtrics online study. The study received ethical clearance from Saint Mary’s University (REB#20-110). This study is part of a larger dataset, which includes a previously published study containing questions on strategies used to quit vaping and relapse triggers experienced by ex-vapers [23]. Ethics approval was also obtained from Qatar University to allow for the participation of one of the authors in the study (QU-IRB 157/2025-EM). Participants had to be from Nova Scotia, 16–24 years old, have a history of sustained nicotine-based product use (three months, where participants vaped a minimum of one day per week in each week during those months), and be currently abstinent from vaping for at least a month. Participants were entered into a draw for one of three gift cards, each valued at USD 100, and each received USD 10 for fully completed surveys. If participants consented to the study, they then answered demographic, past vaping frequency, quit attempts, and open-ended questions. To get a better understanding of quitting behavior, participants were asked two questions: “What was most helpful in quitting vaping?” and “What was least helpful in quitting vaping?” We conducted a content analysis to determine the attitudes and behaviors related to quitting amongst the participants. Three coders had an initial review of the data, and two coders met to discuss emerging themes, the naming of themes, and sub-themes. This allowed for codes to be defined and redefined as needed and capture all emerging themes throughout the data. This process was reviewed by the third coder. After coming to an agreement on a working code list, the coders returned to the data and began coding each response with between 1 and 4 theme and a corresponding sub-theme. In addition to coding, coders were asked to make a note of responses that were vague or where participants appeared to misunderstand the question. Inter-rater reliability (IRR) was calculated. After the initial round of coding, the IRR for Q1 was 61.7% and 78.1% for Q2. The coders worked through any coding discrepancies until both IRRs were 100%. The data was then categorized by age and labeled as youth (16–18) and YAs (19–24) to analyze the themes seen throughout the age groups.

## 3. Results

A total of 281 ex-vapers answered at least one of two questions. Of these, 269 answered Q1 and 279 answered Q2. Out of 548 total responses, 105 were removed due to vagueness or misunderstanding (35 Q1; 70 Q2). After removal, there were 233 responses to Q1 (101 youth; 132 YAs) and 208 responses to Q2 (94 youth; 114 YAs). Details on the characteristics of participants are included in Table 1. On average, prior to quitting vaping, youth and young adults vaped around 6 days per week, with a slightly lower average number of episodes per day for youth (around 28 episodes) relative to young adults (29 episodes), yet youth reported a higher number of puffs per episode on average (8 puffs) relative to young adults (approximately 7 puffs). Both youth and young adults had attempted to quit three times on average before achieving their current abstinence status of 30 days of quitting or longer. The sample was predominantly White for both age groups (youth: 87.5% versus young adults: 85.1%) and mostly consisted of females, yet the percentage of females was evidently higher for youth (72.5%) versus young adults (66.5%). Around 80% of youth and young adults lived in urban areas with similar current employment—60.8% of youth were currently employed relative to 62% of young adults.

### 3.1. What Was Helpful in Quitting

**Figure 1:**
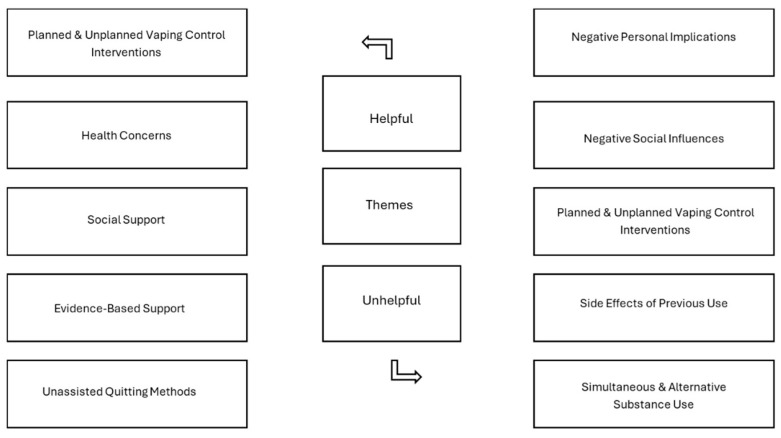
Helpful and unhelpful themes.

**Figure 2:**
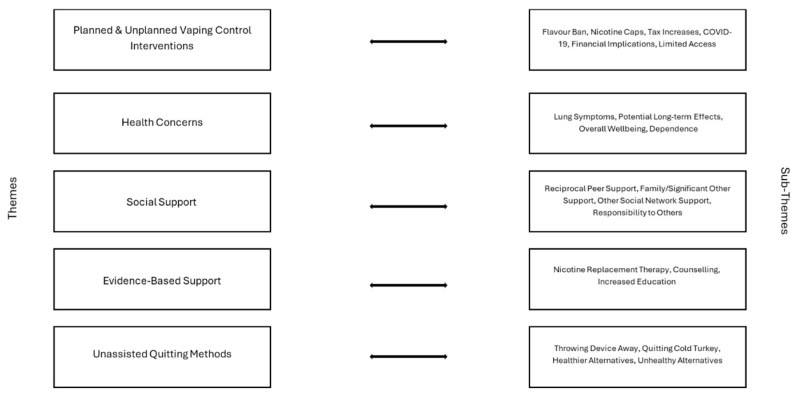
Helpful themes and sub-themes.

When participants were asked to reflect on what was helpful in supporting them while quitting vaping, five themes and twenty-one sub-themes were identified (see Figure 1 for themes; see Figure 2 for sub-themes). Across the two age groups, the themes were consistent: planned and unplanned vaping control interventions (*n* = 48, 21%), health concerns (*n* = 41, 18%), social support (*n* = 71, 30%), evidence-based support (*n* = 16, 7%), and unassisted quitting methods (*n* = 57, 24%).

#### 3.1.1. Planned and Unplanned Vaping Control Interventions

The theme planned and unplanned vaping control interventions was coded a total of 48 times throughout the sample. From those 48 responses six sub-themes were identified: flavor ban (*n* = 14, 29%), nicotine caps (*n* = 2, 4%), tax increases (*n* = 1, 2%), COVID-19 (*n* = 13, 27%), financial implications (*n* = 6, 13%), and limited access (*n* = 12, 25%).

When participants spoke about government-induced restrictions on flavor bans, nicotine caps, or taxation increases, this was coded as a *planned vaping control intervention*. These policies put in place by the government served as a needed intervention to limit access to these products for both youth and YAs, which were supportive in their efforts to quit.

Additionally, many participants spoke about what we coded as *unplanned vaping control interventions*. This included the impacts of COVID-19, financial implications, and limiting access to other vapers. Across both age categories, the benefits of the lack of socialization during COVID-19 was noted: “As quarantine happened, being away from other people such as peers who did it made me stop.” Participants also identified personal finance implications that served as an intervention. The motivator for participants in both categories was either the savings that occurred after quitting: “I have saved a lot of money since I’ve quit, it’s awesome!” Or the lack of ability to afford the products while vaping: “I just genuinely have no money in my bank account, so it wasn’t possible for me to purchase nicotine products.”

#### 3.1.2. Health Concerns

The theme *health concern* was used to code participants responses that referenced health-related concerns as the primary motivator for quitting vaping. This theme was coded 41 within the 233 responses. It included sub-themes consisting of lung symptoms (*n* = 11, 27%), potential long-term effects (*n* = 9, 22%), overall well-being (*n* = 19, 46%), and dependence (*n* = 2, 5%). Some participants reflected on their own health concerns: “I continued to tell myself it was for my health, and that just because it was “cool” my health and feeling normal was cooler.” Other participants reflected on how witnessing the health implications of others served as the motivation they needed: “My grandma having many health problems due to cigarettes.” Within the youth category, participants discussed their concerns regarding lung function which directly impacted their sports performance. One participant stated: “Track and field was what motivated me. I wanted to be the best. When I was nicotine-free for 2 weeks, I got a nice energy boost and didn’t feel as tired.”

#### 3.1.3. Social Support

The *social support* theme was used to capture both the social support offered by those supporting participants with their own desires to quit and the external pressure applied by participants’ social networks for them to quit. Social support was identified as the primary supportive mechanism to quit in 71 responses throughout the sample. The sub-themes used to capture this were, reciprocal peer support (*n* = 28, 39%), family/significant other support (*n* = 27, 38%), other social network support (*n* = 7, 10%), and responsibility to others (*n* = 9, 13%). In the youth category, participants reflected on the role family support offered them in their quitting journey, “My mom and dad were really patient with me, and they stopped smoking around me. My mom was really concerned for my health and suggest (ed) I quit….” In the YA category, participants shared the benefits of having the support of their peers “I talked to my friends every time I felt like wanting nicotine again.”

Participants also shared that as the popularity of vaping decreased in schools this became a motivator for them to follow suit. One youth stated: “I began to see less people doing it at my school, and all of my friends either quit or didn’t have an addiction in the first place, so it felt pretty bad then knowing I still did.” Responses in this category also revealed that pressure from a family or friend could serve as a motivator for quitting; a YA stated: “I went cold turkey while visiting my parents’ house. They’re very anti-smoking….” Additionally, in the YA category, responses showed participants having a growing concern for the impact their vaping would have on their children as evident from the following quote: “I was concerned about the effects vaping would have on my son when he was born.”

#### 3.1.4. Evidence-Based Support

The theme *evidence-based support* was used to capture the responses that stated participants were using scientifically backed support as a method of successfully quitting; it was coded for 16 times. The sub-themes included nicotine replacement therapy (NRTs) (*n* = 9, 56%), professional counselling/support programs (*n* = 3, 19%), and increased education levels (*n* = 4, 25%). Different forms of NRTs were found across the sample, including nicotine patches, gum, and self-monitored nicotine tapering: “With the vaping I was usually using 50–60 mg of nicotine juice and I had to slowly drop it by buying them illegally after the ban was imposed and slowly dropping my nicotine level over the course of a month down to 12 mg then after a bit quit it.” NRTs and tapering worked for many participants in the sample, but for others they found that increased knowledge and understanding of the impacts of vaping was their primary motivator to quit: “Researching and seeing how badly it could affect my lungs” and similarly: “Articles on the negative impacts of vaping found on the news or internet.” A youth participant also shared that they were able to access professional support through a teen health center, that allowed them to better understand the consequences of vaping: “Counselors helped (teen health center) I just told myself the benefits of quitting and how much healthier I would be without it.”

#### 3.1.5. Unassisted Quitting Methods

*Unassisted quitting methods* was the fifth and final theme used to capture participants’ responses to helpful supports in their quitting journey; it was coded a total of 57 times throughout the 233 responses. This theme captured the mechanisms participants could individually rely on to quit without intervention or support from others. The sub-themes used for this theme included throwing the device away (*n* = 5, 8.8%), quitting cold turkey (*n* = 28, 49.1%), swapping for healthier alternatives (*n* = 14, 24.6%), and swapping for unhealthy alternatives (*n* = 10, 17.5%).

Both age categories reflected on using alternative coping mechanisms as a way of supporting them while trying to quit vaping. For some participants, this included swapping vaping for healthier alternatives such as exercise. However, some participants chose unhealthy alternatives as a mechanism as well. One youth participant used pop as a mechanism: “I drank a bunch of pop to help me not vape.” Another stated, “Whenever I’d get a craving I’d just go eat candy or something.” The YA participants were more likely to use coffee: “I ended up drinking more coffee for a while, basically whenever I had a craving.”

Responses under this code also highlighted the importance of participants deciding for themselves to quit: “No one really help me quit vaping because it was my own idea and I believe when someone tries to pressure you to quit smoking it just makes you want to smoke more.” Several participants spoke about how, after they decided they were going to quit, they simply walked away cold turkey or were able to just throw their devices out: “I decided not to opt for any help and just quit cold turkey. Selling my vape + accessories made it easier not to fall back into bad habits.” Also noted under this theme was the need to decrease other substance use to successfully quit vaping. Two participants spoke about how decreasing the use of other substances, specifically alcohol, lowered their desire to vape while they were trying to quit: “I also had to stop drinking for a few weeks after quitting because my cravings were more intense when drinking.”

### 3.2. What Was Least Helpful in Quitting

**Figure 3:**
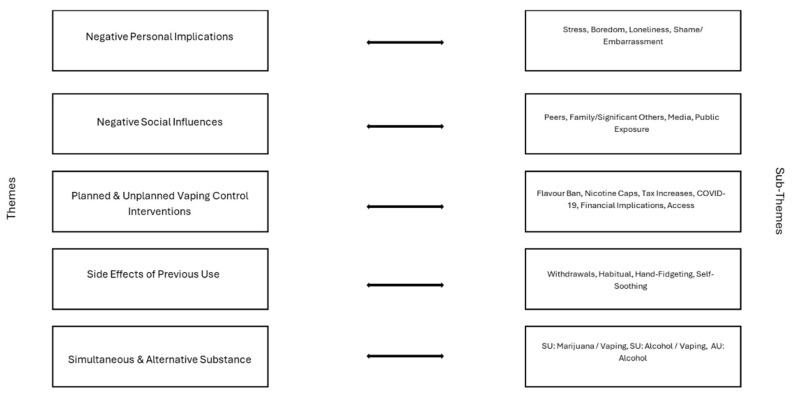
Unhelpful themes and sub-themes.

Participants were asked to share what was not supportive to them in their efforts to quit vaping. After the analysis, there were five themes identified and twenty-one sub-themes. The themes are identical across both age categories. The themes include negative personal implications (*n* = 27, 13%), negative social influences (*n* = 131, 63%), planned and unplanned vaping control interventions (*n* = 27, 13%), side effects of previous use (*n* = 8, 4%), and simultaneous and alternative substance use (*n* = 15, 7%). (See Figure 1 for themes; see Figure 3 for sub-themes.)

#### 3.2.1. Negative Personal Implications

Responses coded for *negative personal implications* were reflections of personal habits or struggles that derailed the participants’ efforts to quit. This was coded as the primary challenge to quitting 27 times throughout the sample. The sub-themes included stress (*n* = 13, 48%), boredom (*n* = 11, 41%), loneliness (*n* = 1, 4%), and shame/embarrassment (*n* = 2, 7%). Stress because of family, school, isolation, or work derailed several participants from their efforts to quit: “I was isolated from my friends and feeling stressed” and: “My family was not helpful as they are one of the stressors for my vaping, as well as my job at the time was not helpful.” Additionally, participants stated that boredom often led to their continued vaping: “I would find myself bored and automatically wanted to reach for a vape and start using it.”

#### 3.2.2. Negative Social Influences

The *negative social influences* theme emerged from responses that depicted participants being pressured back into vaping. It was the most prominent theme throughout the sample, being coded for 131 times out of 208 responses. Within those 131 responses, the sub-theme coded the most was pressure from peers (*n* = 71, 54.2%) followed by public exposure (*n* = 40, 30.5%), family/significant others (*n* = 14, 10.7%), and the media (*n* = 6, 4.6%).

Youth reflected on the difficulties of being pressured by their peers: “In high school almost everyone does it and it’s hard not to with all the peer pressure.” Participants also reflected on the balancing of peer pressure and internal disappointment: “Friends peer pressuring me in social settings, but I just knew that when I would be alone again it would be very disappointing….” Similarly to how the youth reflected on peer pressure within schools, the YA participants shared experiences of being pressured by co-workers: “Co-workers definitely weren’t helpful. Many of them smoke. It took a while for people to stop asking me to go out for a vape.”

It also captured statements participants made illustrating how exposure to other vapers was not helpful in their efforts to quit. The youth reflected on how constant exposure made vaping products too accessible for them to quit: “A lot of family and friends use vape around me, and it was always accessible for me so that was definitely hard.” Additionally, the ease of doing it around school property provided kids with too much temptation: “A lot of my peers vape, and we can do it literally 39 s away from school property.” Similar experiences were shared from the YA participants in the context of exposure at universities: “Coming back to school after covid has made it tempting, because it’s everywhere.”

#### 3.2.3. Planned and Unplanned Vaping Control Interventions

The theme *planned and unplanned vaping control interventions* was found in both the helpful and unhelpful participant responses. For the responses illustrating what was not helpful, it was coded 27 times. This code was used to capture sub-themes that included reflections on the change in government policies such as flavor bans (*n* = 8, 29.6%), nicotine caps (*n* = 3, 11.1%), and tax increases (*n* = 1, 3.7%). It also captured sub-themes regarding COVID-19 (*n* = 5, 18.5%), financial implications (*n* = 1, 3.7%), and access to products (*n* = 9, 33.3%).

Responses that reflected on how the increased regulation did not help them quit vaping were coded using the sub-themes: flavor bans, nicotine caps, or tax increase. Participants spoke about how, even with the change in regulations, flavored products and higher nicotine juice were still easily accessible. Participants shared strong feelings in response to the increased regulations: “The flavor ban did nothing for me and in my opinion, it makes it harder for smokers to transition to vaping before quitting, which is what I did.” Not only did they feel it was not supportive in their quitting journeys, but it was eliminating their ability to make their own choices as consenting adults: “I am a low income, high stress individual who saw the flavor ban and impending per-milliliter taxation and had to make a difficult decision to stop doing something that I enjoyed as a consenting adult.” Participants in the youth category also shared feelings of disdain towards the change in regulation sharing that: “The ban on flavors made me want to keep going out of spite.”

Participants reflected on how, despite the desired intervention, accessing products did not become an issue for them. Youth participants spoke about how the ease of accessing products, even after the change in regulation, made continuing to vape easy: “Someone I know had very easy access to vaping products, and still does. I got them for very cheap, which did not help.” YAs shared similar experiences post regulation change, as backdoor channels still allowed them access to the products they wanted: “I am originally from [another province], so I had ways to get vaping products from there this summer. This made the ban on flavored and high nicotine products useless.”

One of the lesser themes that emerged from the data was the financial implications, or the lack thereof. Participants spoke about their ability to still access cheap products: “People continued to sell high nicotine and flavored juice for very cheap ‘under the table’, so for me and my peers it became more accessible and cheaper to get.” The data also revealed discussion related to how the value of the product outweighed the increase in price and tax: “I don’t care how much it costs or if it’s taxed, I’d still buy it”, which lead to financial implications not being a helpful motivating factor to quit.

#### 3.2.4. Side Effects of Previous Use

Participants in both age categories spoke about how experiencing *side effects of previous use* was detrimental to their efforts to quit (*n* = 8, 4%). The sub-themes included withdrawal (*n* = 2, 25%), habitual (*n* = 1, 12.5%), hand-fidgeting (*n* = 2, 25%), and self-soothing (*n* = 3, 37.5%). The analysis showed that for some participants, it was more about the vaping itself and less about the impacts of nicotine: “How fun and addicting the motion of vaping is. Not just the nicotine.” Similarly, they shared that vaping was more an avenue of relaxation than an addiction to the substance, “I always have the urge to do something when I’m sitting down, so vaping made my hands occupied while I was watching tv or doing homework.”

#### 3.2.5. Simultaneous and Alternative Substance Use

Participants reflected on the simultaneous use of vaping and other substances derailing their efforts to quit or picking up vaping as a mechanism of quitting another substance such as alcohol. This theme was coded 15 times throughout the sample (7%). The sub-themes included simultaneous use with marijuana (*n* = 2, 13%), simultaneous use with alcohol (*n* = 12, 80%), and alternative use to consuming alcohol (*n* = 1, 7%).

Within the youth category, the analysis relieved that vaping was used as an alternative to other substances. One youth participant shared that they turned to vaping instead of drinking: “I had access to it and I was really stressed out at the time so I went to vaping instead of drinking.” Whereas the YAs experienced more of the simultaneous substance use pressure: “Also, being drunk seemed to have triggered the need to vape” and “drinking definitely doesn’t help either, as I always vaped more when drunk and that still brings an instant craving.”

## 4. Discussion

This study sheds light into the importance of asking “what” and “who” was helpful/not helpful in supporting quit attempts. It also unravels important differences between youth and YAs with respect to these factors in Nova Scotia, Canada, where a jurisdiction that implemented vaping control policies occurred shortly prior to the collection of data for this study. Overall, when asked about what youth and YAs found helpful for quitting vaping, five themes emerged—*evidence-based support*, *social support*, *health concerns*, *unassisted quitting methods*, and *planned and unplanned vaping control interventions*. When asked about the contrary, the youth and YAs listed *negative personal implications*, *negative social influences*, *planned and unplanned vaping control interventions*, *side effects of previous use*, and *simultaneous*/*alternative substance use* as unhelpful factors that derailed their quit vaping journeys. Details of the sub-themes and differences in helpful and unhelpful factors between youth and YAs are discussed below and in relation to the larger literature.

First, under helpful factors, for the planned and unplanned vaping interventions theme, youth and YAs both identified *limited access* as a sub-theme that drives quitting. COVID-19 regulations, flavor bans, and nicotine caps were other sub-themes that emerged, suggesting that regulation/policy, whether unplanned, due to COVID-19 restrictions, or planned (e.g., nicotine cap), is effective in helping youth and YAs quit vaping. This is logical given that COVID-19 restrictions forced youth to quarantine where they were less likely to vape as many tend to vape discretely, such as vaping in school bathrooms [25].

For youth and YAs, health concerns, which encapsulate the sub-themes of overall well-being and lung function as well as the fear of unknown long-term effects, were noted as helpful factors for quitting. In the media, long-term harms have been the main messaging line of social marketing campaigns for health advocacy in Canada [26]. This seems to be a powerful message to encourage people to quit vaping for both age groups.

Social support, whether through reciprocal peer support, family, significant others, or social networks, and responsibility to others was another important helpful theme that emerged from participant responses. This finding underscores the importance of social circles in vaping cessation [27,28]. Youth mentioned family, responsibility to others, and reduced popularity in school settings as helpful social support factors for quitting. While most of the past studies focused on how parents can help prevent youth smoking (e.g., [29]), the current study adds to that literature by revealing that parental support can help youth quit vaping. Responsibility to others is likely important for youth due to an increase in school-level policies against vaping [19]. YAs in this study mentioned friends/support by other peers as sources of social support. Given that the past literature provided support for buddy-based app support for smoking cessation [30], the same could be useful for vapers.

Evidence-based support was reported as a helpful factor for quitting by ex-vapers in this study. This is in line with the past literature which has shown that NRTs, counseling, and other similar approaches have shown value in helping vapers quit [31,32]. Similarly, unassisted methods such as quitting cold turkey or adopting healthier alternatives were used by ex-vapers in this study to quit vaping, which is consistent with the past literature [33].

With respect to what, or who, was not helpful in supporting the ex-vapers’ quit journeys, one main factor was negative personal implications exemplified in struggles to quit, the perpetuation of boredom, and stress due to feeling socially isolated because of vaping. The past literature has shown that boredom and stress are reasons for vaping [12]; the current study shows that such stress and boredom also makes it difficult to quit vaping. Youth and YAs are particularly vulnerable because of the stress and boredom associated with the transition to college, university, or full-time employment, and independent living relative to youth [34].

Negative social influences was another unhelpful factor in the journey to quit vaping. This took the form of social pressure from friends and other people who vape in one’s social circle, and seemed to make it difficult for ex-vapers to quit. Family and significant others who vape were of secondary importance when it comes to negative influence. Furthermore, some noted that media ads had a negative influence as they may have been deterred from quitting vaping due to the imposing nature of the ads’ messages which called on the viewer to quit vaping. This likely refers to Health Canada’s “Consider the Consequences of Vaping” campaign against vaping [26].

While planned and unplanned vaping control policies were listed as helpful factors by some participants, they were also perceived negatively as a factor for quitting. Some participants felt that these policies discouraged quitting via inciting rebellion to increased regulation. This is likely related to the feeling of infringed liberty regarding the use of desired varieties of flavors and concentrations of nicotine. Others mentioned their perceived ineffectiveness of the policy—due to the ease of access of vaping products despite regulations, for example. In all likelihood, the participants may be referring to accessing flavored products from neighboring provinces. Despite these observations, evidence from past studies demonstrates that vaping regulations are associated with less vaping [35], perhaps highlighting that a negative perspective about policies is not necessarily representative of actual ex-vaper behavior.

Some participants spoke about withdrawal as a factor that deterred past attempts to quit vaping due to the association of vaping with relaxation and enjoying the hand-to- mouth movement. This is consistent with past findings that have touched on the enjoyment vapers get from watching the vape clouds that are emitted from them [12]. This study shows that this enjoyable aspect is also a deterrent to quitting vaping.

Simultaneous drug/substance use was a barrier for some ex-vapers during their quit journeys, but this was more of an issue for YAs, especially when it comes to alcohol. This finding is expected as YAs are of a legal age to purchase other drugs and have reported drugs and alcohol as a relapse factor in the past literature [23]. Steps towards encouraging polysubstance treatment may therefore be essential to YAs.

## 5. Limitations

There are several limitations in this study. The study used an open-ended survey rather than semi-structured interviews, which limits the depth of responses and the opportunity to ask probing questions for clarification. Data was removed if participants seemed to not understand the question, meaning that interviews may have helped the participant clarify their responses. However, we received a large number of high-quality responses to the open-ended survey which may compensate for this limitation. This study also used self-report measures. There is always the possibility that participants were not honest related to their current vaping status. There is also the possibility that participants were limited by self-awareness related to the factors that were helpful or not. Future research using a longitudinal or diary study would be useful. For example, in a diary study, when participants recognize a behavior that may be helpful or not, they could record it instantaneously, and being able to track this over a long period with a group of participants would eliminate concerns regarding cross-sectional designs. Finally, participants may have provided favorable responses (reflecting potential social desirability bias) and not have recalled their quitting journey accurately (reflecting potential recall bias issues).

## 6. Conclusions

Youth and YAs share common factors for the facilitators and barriers in their quit journeys. This study suggests that focusing on awareness about lung health and well-being may be promising avenues to support youth. Increasing awareness about the role of social support and messaging about general health concerns and addiction seem to be important for YAs’ quit journeys. It appears that increasing the cost of e-cigarettes facilitates quit journeys for YAs. Training in managing social pressure as a barrier to quitting may be important for youth, while stress management and polysubstance treatment for YAs are likely to reduce impediments to quitting vaping. Policies, such as flavor ban and nicotine caps seem to have factored into the decision of some participants to quit vaping. Overall, there are many strategies to support quitting, but age needs to be a factor that is considered when designing programs.

## Figures and Tables

**Table 1:** Characteristics of sample before quitting.

Variable	Youth (*N* = 120), *M* (*SD*)	YAs (*N* = 161), *M* (*SD*)
# of vaping days per week	6.34 (1.29)	6.65 (1.03)
# of vaping episodes per day	27.75 (30.16)	29.40 (30.42)
# of puffs per episode	8.01 (6.47)	6.75 (5.01)
Quit attempts	3.25 (2.47)	3.05 (2.17)
	N (%)	N (%)
Race		
White	105 (87.5)	137 (85.1)
Other ethnicity	15 (12.5)	24 (14.9)
Gender		
Female	87 (72.5)	107 (66.5)
Male	33 (27.5)	54 (33.5)
Housing		
Urban	96 (80)	130 (80.7)
Rural	24 (20)	31 (19.3)
Employed		
Yes	73 (60.8)	100 (62.1)
No	47 (39.2)	61 (37.9)
Total	120	161

## Data Availability

Data is not readily available. Due to the sensitive nature of the data, researchers declared in the informed consent that the data will be available for five years from the start date of the study. Further inquiries can be directed to the corresponding author.

## Abbreviations

The following abbreviations are used in this manuscript:

YA	Young Adults
NS	Nova Scotia
IRR	Inter-rater Reliability
NRT	Nicotine Replacement Therapy
